# NOD1: a metabolic modulator

**DOI:** 10.3389/fendo.2024.1484829

**Published:** 2025-01-21

**Authors:** Ruobing Tang, Chunguang Xie, Xiyu Zhang

**Affiliations:** ^1^ Hospital of Chengdu University of Traditional Chinese Medicine, Chengdu, Sichuan, China; ^2^ TCM Regulating Metabolic Diseases Key Laboratory of Sichuan Province, Chengdu, Sichuan, China; ^3^ Department of Endocrinology, Hospital of Chengdu University of Traditional Chinese Medicine, Chengdu, Sichuan, China

**Keywords:** NOD1, metabolism, endocrine, inflammation, immunity

## Abstract

Nucleotide-binding oligomerization domain 1 (NOD1) is an intracellular pattern recognition receptor that detects injury signals and initiates inflammatory responses and host defense. Furthermore, NOD1 serves as a metabolic mediator by influencing the metabolism of various tissues, including adipose tissue, liver, cardiovascular tissue, pancreatic β cells, adrenal glands, and bones through diverse mechanisms. It has been discovered that activated NOD1 is associated with the pathological mechanisms of certain metabolic diseases. This review presents a comprehensive summary of the impact of NOD1 on tissue-specific metabolism.

## 1 Introduction

Nucleotide-binding oligomerization domain-1 (NOD1), a member of the NOD-like receptor family, is an intracellular immunoregulatory protein (1, 2). NOD1 is widely distributed in antigen-presenting cells and epithelial cells (3). It recognizes damage signals and triggers an inflammatory response to mediate host defense (4). It has been demonstrated that NOD1 and its downstream pathways activated in the adipose tissue of individuals diagnosed with metabolic syndrome, and gestational diabetes mellitus, as well as those who are overweight or obese (5–7). Additionally, there is a significant increase in NOD1 expression in the myocardium of individuals diagnosed with type 2 diabetes mellitus (8). These studies indicate that NOD1 may be involved in metabolic processes and could play a role in metabolic diseases. This article provides a comprehensive review of the impact of NOD1 on metabolism across various tissues and organs, highlighting its potential significance in understanding metabolic disorders. Furthermore, it introduces the potential for NOD1 to serve as a risk assessment marker for metabolic diseases.

## 2 NOD1

The NOD1 protein consists of three domains. The C-terminus contains leucine-rich repeats, which are capable of recognizing specific ligands. The central nucleotide-binding domain exhibits ATPase activity and undergoes conformational changes upon ligand activation, thus promoting the oligomerization of NOD1. The N-terminal contains a caspase-activation-and-recruitment-domain (CARD), which is used to recruit downstream effectors (9–11).

NOD1 is a classical cytoplasmic pattern recognition receptor capable of recognizing pathogen-associated molecular patterns (1, 12). NOD1 specifically recognizes the peptidoglycan (PGN) fragment γ-D-glutamyl-meso-diaminopimelic acid (iE-DAP) on the bacterial cell wall (1, 13). Additionally, NOD1 was found activated during viral, fungal, and parasitic infections. For example, the hepatitis C virus synthesizes dsRNA through a nonstructural protein 5B RNA-dependent RNA polymerase. dsRNA interacts with NOD1 to promote its activation (12). The parasitic nematode *L. sigmodontis* has been identified as a host of the intracellular symbiont Wolbachia, which possesses all the necessary enzymes for synthesizing lipid II, the precursor of bacterial peptidoglycan. Consequently, *L. sigmodontis* is capable of activating NOD1 (14). Furthermore, there have been reports of human cytomegalovirus (15), Aspergillus fumigatus (16, 17), *Trypanosoma cruzi* (18), and *Plasmodium berghei (* 19) activating NOD1. However, the precise mechanism of their activation remains unclear. In addition to recognizing pathogen-associated molecules, NOD1 is also capable of recognizing danger-associated molecular signals. A deficiency in NODs has been observed to attenuate the inflammatory response by DTT, an endoplasmic reticulum (ER) stress inducer (20). Peptidoglycan-independent Brucella abortus has been demonstrated to induce ER stress through the type IV secretion system, thereby activating the NOD1/RIP2 signaling pathway (20). The aforementioned evidence suggests that ER stress may serve as one of the signals for NOD1 activation (20). Interestingly, thapsigargin, an ER stress agonist and a sarcoplasmic reticulum Ca²^+^ ATPase inhibitor has been demonstrated to induce ER stress by mediating calcium influx. It subsequently leads to the internalization of peripheral micro-peptidoglycan and activates cytoplasmic NOD1. Indicates that calcium influx contributes to NOD1 activation (21). Further investigation is required to elucidate the independent role of calcium influx and ER stress in NOD1 activation. Moreover, NOD1 detects aberrant expression of small Rho GTPases triggered by perturbations in host cell function caused by bacterial virulence factors (22). In this way, NOD1 identifies the pathogenic factors of *Salmonella enterica* (23) and *Shigella flexneri* (24). Recently, it has been found that the activated small Rho GTPase Rac1 activates intracellular NOD1 during hematopoietic stem and progenitor cell differentiation. NOD1-mediated “Developmental Inflammation” induces hematopoietic stem and progenitor cells to differentiate into hemogenic endothelium (25). It again demonstrates the ability of NOD1 to sense intracellular perturbations and signals from small Rho GTPases, suggesting that NOD1 influences the physiological function of cell fate. However, further study is needed to determine whether there is selectivity in NOD1 recognition of such signals.

After NOD1 is activated, it interacts through the CARD domain to bind with receptor-interacting kinase 2 (RIP2) receptors to form protein complexes (26). E3 ligases, such as inhibitors of apoptosis proteins (IAP) like c-IAP1, c-IAP2, and XIAP (27, 28), mediate the conjugation of RIP2 to the K63 ubiquitin chain (29). Polyubiquitylated RIP2 recruits TAK1 and interacts with the IκB kinase (IKK) subunit NEMO (IKKγ) to recruit the IKK complex. The IKKs are subsequently activated by TAK1 (29). In addition, the formation of the TAK1 kinase complex leads to the activation of MKK6, which in turn activates mitogen-activated protein kinase. Finally, it triggers downstream signaling pathways including nuclear factor kappa B (NF-κB) and mitogen-activated protein kinase to promote cytokines release (9, 26, 30). Another signal pathway has identified in epithelial cell lines. NOD1 recruits RIP2, binds to TNF receptor-associated factor 3, and activates the IFN regulatory factor 7. Induces the production of IFN-β and activates IFN-stimulated gene factor 3 for type I IFN signaling. This pathway has a mucosal protective function (31).

**Figure 1:**
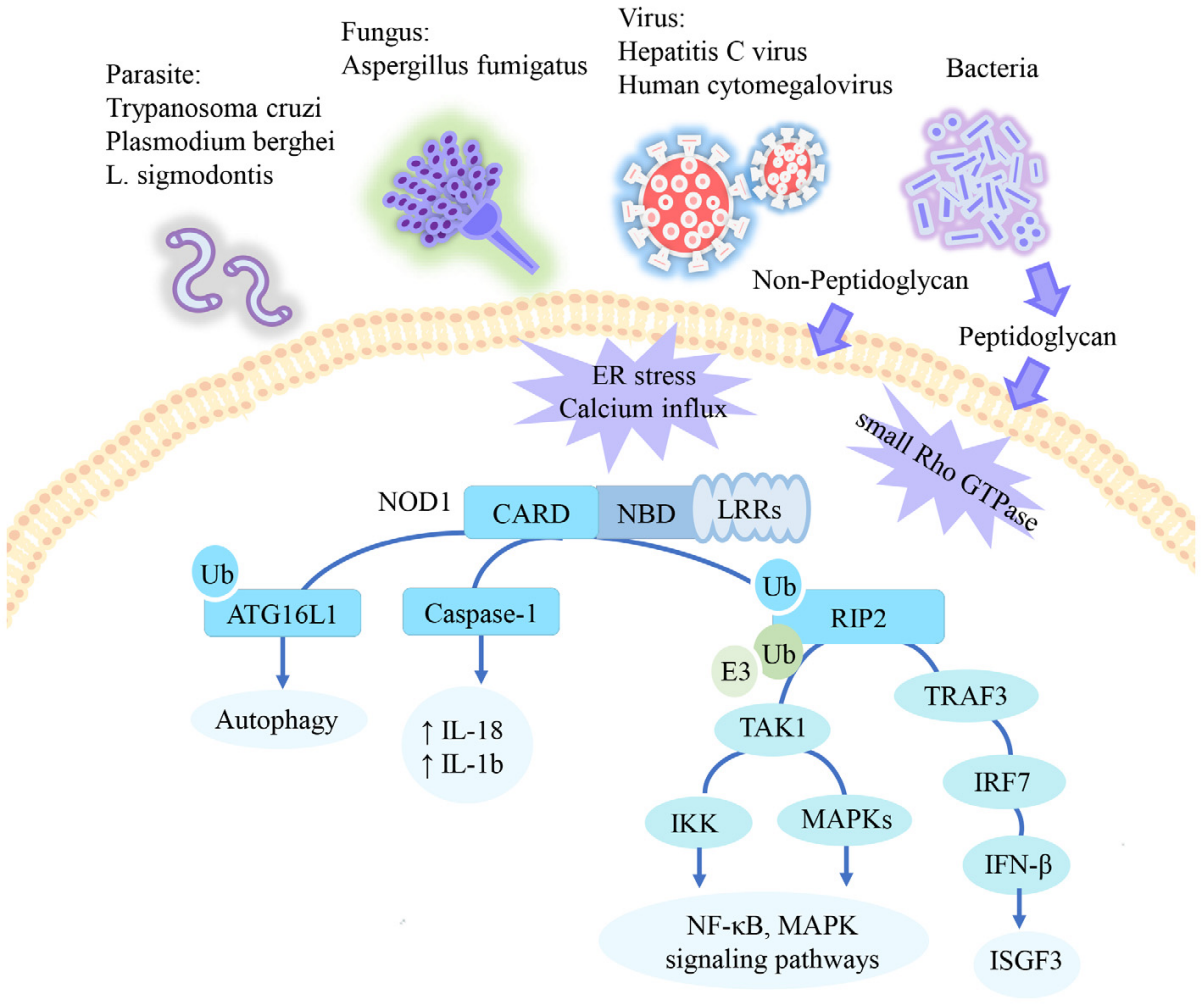
The signaling pathway of NOD1. NOD1 specifically recognizes the peptidoglycan fragment iE-DAP in pathogens. In addition to peptidoglycan, NOD1 has also been reported to be activated by parasite, certain viruses, and danger signals. Activated NOD1 undergoes self-oligomerization and recruits downstream effector proteins. NOD1 recruits RIP2, which undergoes multiple ubiquitination and phosphorylation to mediate downstream activation of the NF-KB and MAPK pathways. In epithelial cells, this pathway mediates type 1 IFN signaling. Additionally, ATG16L1 and Caspase-1 have been reported to be activated through NOD1 recruitment, promoting apoptosis and inflammation respectively. ↑= increased.

Independent of the NOD1/RIP2 pathway, NOD1 induces autophagy-related16-like 1 (ATG16L1) translocation to the plasma membrane in response to bacterial infection. ATG16L1 exerts a negative regulatory influence on the assembly of the NOD1/RIP2 complex (32, 33). Furthermore, ubiquitin competitively regulates the binding of NOD1 to RIP2 and ATG16L1 (34). Additionally, NOD1 interacts with caspase-1 to mediate IL-18 maturation and IL-1b secretion (35, 36). Previous research has shown that NOD1 enhances procaspase-9 activation and caspase-9-mediated apoptosis (9). However, it remains unclear whether this process is independent of NOD1/RIP2. The signaling pathway of NOD1 summarized in **Figure 1**.

## 3 NOD1, commensal microbiota, metabolism

In mammals, a great deal of microbiota coexist with host cells, and their status is closely related to host health (37). In the intestine, a barrier system consists of the intestinal epithelium and mucosa (38). This system separates the commensal bacteria and metabolites within the intestinal tract from the body’s internal environment effectively reducing potential disease risks (39). However, under conditions such as high sugar intake, high-fat consumption, obesity, and inflammation, an imbalance in intestinal flora occurs along with increased intestinal permeability. Consequently, intestinal bacteria and metabolites are released into the bloodstream, causing chronic low-grade inflammation and metabolic dysfunction within target organs (40–42).

NOD1 is a protector of intestinal barrier function (43, 44). It monitors danger signals in intestinal epithelial cells and mediates the immune response to pathogen invasion (22). NOD1-deficient mice exhibit increased levels of inflammatory mediators in the intestines, altered immune characteristics (such as decreased expression of NOD2, secretory mucin, host defense peptide, and keratinocytes), and enhanced intestinal permeability (43, 45). The impact of NOD1 on the abundance of gut microbiota remains controversial. A previous study did not find evidence that NOD1 deficiency affects the microbiota in the ileum and cecum of mice (45). However, a recent study revealed an increased abundance of Parabacteroides, Rikenella, Prevotella, and Helicobacter in the intestines of NOD1^-/-^ mice (46). It suggests that while maintaining intestinal homeostasis, NOD1 may regulate the composition of intestinal flora. The discrepancy between these findings may attributed to differences in approaches used for microbiota analysis in the two studies.

The specific ligand of NOD1, iE-DAP, is derived from PGN. It has been demonstrated that the majority of circulating PGN originates from the host microbiota (47). The serum levels of PGN in specific pathogen-free mice are approximately 0.18-0.3 µg ml^-^¹, while in germ-free mice, they are undetectable (47). It has been established that the intestine harbors a vast array of colonizing microorganisms (48). Intestine exists an iE-DAP production pathway. The iE-DAP fragments are cleaved from PGN by lysozyme secreted by intestinal Paneth cells (49). In conclusion, iE-DAP primarily derived from host microbes, with the intestinal tract representing a significant source.

During the process of metabolism, NOD1 receptors present in target tissues recognize ligand signals from the host microbiota, promoting tissue metabolism and facilitating communication between host microorganisms and metabolism. Activation of the NOD1 receptor in pancreatic β cells by intestinal-derived ligands enhance insulin vesicle transport, stimulate insulin secretion, and maintain blood glucose homeostasis (49). Similarly, NOD1 receptors located within the dense core granules of mice adrenal chromaffin cells are capable of recognizing ligands derived from the intestinal tract. They recruit Rab2a, which then mediate the storage and secretion of epinephrine and chromogranin A (50). In addition, the function of intestinal flora in reducing bone cortical thickness and promoting bone resorption (enhanced expression of RANKL and TNFα) in mice is under regulation of NOD1.The bone metabolism in NOD^-/-^ mice remains insensitive to alterations in intestinal flora due to different feeding conditions (51). Overall, while maintaining the intestinal barrier function, NOD1 may contribute to the homeostasis of intestinal flora. Additionally, serving as a sensor, NOD1 detects host microbial signals and is involved in metabolism.

## 4 Activation of NOD1 is regulated by diet

Different diets lead to differences in nutrient intake, which affects the intestinal environment and metabolism (52).

A high-fat diet (HFD) is characterized by high fat intake. It’s a well-known model of abnormalities in glycolipid metabolism (53). The concentration of NOD1 ligands in the circulation showed a significant increase in mice fed an HFD (54). The activity level of NOD1 ligands increased with prolonged exposure to an HFD (41). With the up-regulation of NOD1 expression in adipose tissue, skeletal muscle, liver, and spleen, the expression of inflammatory cytokines also increased (41, 55, 56). HFD may affect NOD1 expression through several factors independently or synergistically with its factors. HFD feeding gradually impairs intestinal barrier function in mice, resulting in enhanced translocation of microbial metabolites, and consequently elevated circulating levels of NOD1 ligands (41, 57). Moreover, it has been demonstrated that HFD contributes to gut microbiota dysbiosis (57–59), characterized by a decrease in Bacteroidetes and an increase in Firmicutes (52). Given the variations in subject, timing of intervention, and composition of HFD, no study has conclusively demonstrated that an HFD directly influences NOD1 by modifying the gut microbiota. However, this dysbacteriosis associated with HFD affects the production of metabolites such as short-chain fatty acids and bile acids (52). Alterations in these metabolites may affect NOD1.

A study found that saturated fatty acid intake was negatively associated with insulin sensitivity in young healthy people with the NOD1 (Glu266Lys) Lys/Lys genotype (60). It suggests that single nucleotide polymorphisms in NOD1, in combination with dietary factors, synergistically increase the risk of metabolic abnormalities. Dietary saturated fatty acids are found in vegetable oils and animal fats. Because of their pro-inflammatory effects, excessive intake has been demonstrated to increase the risk of metabolic disorders (61). Saturated fatty acids (lauric acid) activate the NOD1/NF-κB signaling pathway in human colonic epithelial cells HCT116 to induce inflammation (62). However, NOD1-deficient macrophages are not subjected to inflammatory stimulation by saturated fatty acids (palmitic acid) (54). Both lauric acid and iE-DAP-mediated cellular inflammation were inhibited by polyunsaturated fatty acids (docosahexaenoic acid) (62). These studies support that NOD1 is one of the targets of saturated fatty acid-induced inflammation. Unsaturated fatty acids may be protective against such inflammation. In summary, the dietary structure represented by the HFD model has a notable influence on the expression of NOD1. Dietary fatty acids may be one of the key factors in the involvement of NOD1 in metabolic diseases.

Notably, a study found that, following four weeks of HFD consumption, NOD1^-/-^ mice did not exhibit abnormal glucose tolerance, unlike their wild-type (WT) counterparts (63). It suggests that NOD1 may play a crucial role in the metabolic damage induced by an HFD and that there is a complex interaction between NOD1 and the pathogenicity of an HFD.

## 5 How does NOD1 mediate the metabolism of various tissues

**Figure 2:**
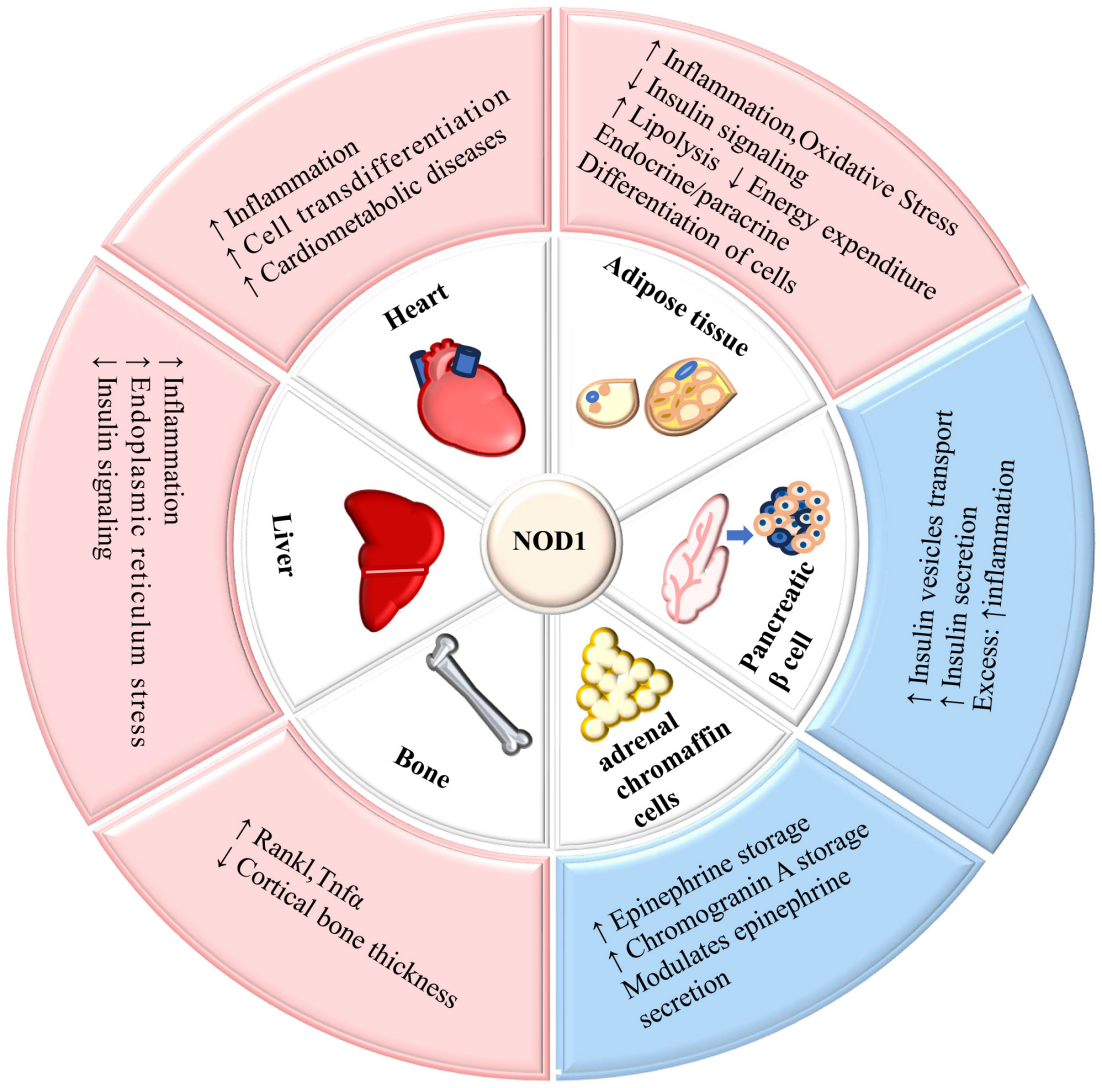
The role of NOD1 in mediating metabolism in tissues. NOD1 is expressed in adipose tissue, liver, cardiovascular tissue, pancreatic β-cells, adrenal glands, and bone, and its activation affects the metabolic function of tissues. ↑= increased; ↓= decreased.

NOD1 is expressed in various tissues including the human heart, lungs, skeletal muscle, liver, kidney, pancreas, spleen, and others (9). The widespread expression of NOD1 significantly contributes to its involvement in the metabolism of multiple organs and tissues throughout the body. The role of NOD1 in mediating metabolism in tissues is summarized in **Figure 2**.

### 5.1 NOD1 and insulin

Insulin is a peptide hormone synthesized and secreted by pancreatic β cells. It is an essential mediator in energy metabolism (64). NOD1 functions as a signal receiver for microbial signals within the pancreatic islets, helping efficient glucose-stimulated insulin secretion (49). Activation of NOD1 in insulin secretory granules by ligands of intestinal microbial origin recruits RIP2 and Rab1a, which then mediates the intracellular transport of the secretory granules, driving them away from the nucleus towards the plasma membrane to complete the secretion of insulin (49). It has been established that NOD1 ligands in normal-diet WT mice are adequate to support insulin secretion. More ligand does not enhance its pro-secretory function (49).

Insulin binds to the insulin receptors in the target cell, activating tyrosine kinases within the receptor. Receptors recruit and phosphorylate tyrosine residues of the receptor substrate, activating downstream signaling and thus building a complex insulin signaling pathway (64, 65). It has been found that NF-κB and MAPK signaling can be crosstalk with insulin signaling (65), at the same time, both signals can activated by NOD1 (4). It establishes a link between NOD1 and insulin signaling. NOD1 can inhibit IRS-1-mediated metabolism-related insulin signaling (5, 64–66). Phosphorylation of JNK in human adipocytes increased after iE-DAP intervention that stimulated phosphorylation of IRS-1 Ser307. Insulin-induced phosphorylation of IRS-1 tyrosine, Akt Ser473, and Akt Thr308 inhibited under the influence of iE-DAP. Ultimately, interfering with insulin signaling reduces insulin-induced glucose uptake in adipocytes (6). In addition to JNK, protein kinases such as ERK1/2 and cytokines such as TNF-α and IL-6 have been reported may be involved in the interference of insulin signaling by NOD1 (5, 6, 67). However, further research is needed to clarify the mechanisms of these NOD1 downstream signaling molecules that affect insulin signaling, to understand the role NOD1 plays in this process.

Insulin regulates the synthesis of glycogen, lipids, and proteins to maintain smooth blood glucose and energy metabolism homeostasis in the body (68). The interference of insulin signaling by NOD1 leads to reduced insulin sensitivity in NOD1-expressing tissues, triggering specific metabolic abnormalities in the corresponding tissues.

### 5.2 NOD1 and adipose tissue

Adipose tissue is essential for energy metabolism, endocrine and immune functions and helps to maintain metabolic balance in the body (69).

#### 5.2.1 Inflammation, oxidative stress, interfering with insulin signaling

NOD1 in adipocytes activates downstream NF-κB signaling, enhances secretion of proinflammatory chemokines, and enhances inflammatory signaling in adipose tissue (66, 67). In addition, Oxidative stress enhanced by activated NOD1 also contributes to adipose inflammation (70). NOD1 activates NOX4, NOX1, and protein kinase Cδ (PKCδ) in adipocytes. PKCδ promoted ROS generation by NOX1 and NOX4 while inhibiting the expression of antioxidant enzymes. It also activates the JNK pathway and NF-κB pathway (70).

Insulin acts in adipose tissue to promote glucose uptake, enhance lipid synthesis, and inhibit lipolysis (64). However, as we explored in section 5.1, NOD1 regulation of the IRS1/Akt pathway hinders insulin signaling, inducing insulin resistance. NOD1 reduces insulin sensitivity in adipose tissue, decreases glucose uptake (6, 67), and increases lipolysis in adipose tissue (70–72). As insulin resistance progresses within adipose tissue, excess lipolysis occurs, which leads to increased production of free fatty acids and ectopic accumulation of fat within the organs that are sensitive to insulin. Results in further exacerbation of insulin resistance (64, 73).

#### 5.2.2 Enhance lipolysis

Lipolysis is a process in which lipase releases fatty acids from fat and generates energy (74). Abnormal lipolysis is associated with the pathogenesis of obesity, type 2 diabetes, non-alcoholic fatty liver, and other diseases (74). Insulin is an inhibitor of lipolysis (75). Insulin inhibits lipolysis by activating phosphodiesterase 3B and suppressing the cAMP and protein kinase A (PKA) (75). When insulin signaling is blocked in adipose tissue, the anti-lipolytic effect of insulin is impaired. However, the activation of NOD1 in adipocytes hinders the insulin signaling pathway, potentially enhancing lipolysis. NOD1 enhances lipolysis both in mouse white adipose tissue (71) and 3T3-L1 adipocytes (70–72). NOD1 regulates the enzymatic activity of hormone-sensitive lipase (HSL) to affect lipolysis (71, 72). HSL is an intracellular lipase widely expressed and primarily involved in adipocyte lipolysis, steroidogenesis, and spermatogenesis processes (76). NOD1 promotes phosphorylation of HSL at Ser563 by PKA to modulate its enzyme activity, and it also facilitates phosphorylation of Plin at Ser517. Which is necessary for efficient lipolysis (72). By mediating the synergistic effects of ERK, PKA, and NF-κB pathways, NOD1 potentiates the HSL-mediated lipolysis program (71).

During the lipolysis process, diacylglycerol accumulates as an intermediate product within cells leading to the activation of PKCδ and subsequent enhancement of oxidative stress levels (70). Meanwhile, PKCδ triggers cascade activation of interleukin-1 receptor-related kinase IRAK1/4, upregulating expression of pro-inflammatory cytokines including IL-1β, IL-18, IL-6, TNF-α, and MCP-1 enhanced inflammation in adipocytes (77).

#### 5.2.3 Endocrine/paracrine

Adipocytes secrete a variety of adipokines, lipids, metabolites, and Exosomal microRNAs. Thus, adipose tissue can regulate the metabolic processes of other metabolic tissues (78). In a study, human hepatocellular carcinoma cells cultured in a conditioned medium from 3T3-L1 adipocytes intervened in advance by NOD1 ligand (iE-DAP) showed lipid accumulation was enhanced (79). It was attributed to the secreted factors such as fatty acids and inflammatory mediators produced by NOD1-involved adipose inflammation and lipolysis, which enhance hepatocyte lipid metabolism. It suggests that NOD1 regulates hepatic lipid metabolism with endocrine/paracrine functions of adipose tissue (79).

#### 5.2.4 Reduce adipocyte energy expenditure

Compared to white fat, brown adipose tissue (BAT) cells are characterized by abundant mitochondria and iron content, as well as multilocular lipid droplets that enhance energy metabolism and thermogenesis in response to cold and other stimuli (80). The classical mechanism of BAT thermogenesis involves the uncoupling of mitochondrial respiration mediated by uncoupling protein 1 (UCP-1) (80). NOD1 inhibits the formation and function of BAT, thereby affecting adipose energy expenditure (81). Activation of NOD1 in mouse embryonic mesenchymal stromal cells C3H10T1/2 and immortalized brown preadipocytes inhibited their differentiation into brown adipose tissue. This inhibition may be attributed to the mechanism where NOD1 induces activation of the NF-κB pathway while inhibiting trans-activation of Peroxisome proliferator-activated receptor gamma (PPARγ) (82). Activated NOD1 suppressed the UCP-1 promoter activity in brown adipocytes, resulting in restricted expression of UCP-1 under both basal and isoproterenol treatment conditions. As a consequence, the oxygen consumption rate of brown adipocytes was reduced in both conditions (81). These findings demonstrate the inhibitory effect of NOD1 on the thermogenic function of BAT.

The impact of NOD1 on thyroid hormone homeostasis may serve as a potential mechanism through which NOD1 suppresses UCP-1 expression in mice (46). After consuming HFD, NOD1-deficient mice exhibited decreased triiodothyronine levels in the liver and BAT, reduced hepatic Pnpla3 expression, and diminished UCP1 expression in the BAT. This led to improved insulin resistance but earlier onset of obesity (46). It has been demonstrated that elevated triiodothyronine levels stimulate lipolysis in animal models, resulting in a reduction in adipose tissue mass (83). The reduction of T3 leads to a deficiency of PNPLA3, an enzyme with lipase activity that is responsible for triglyceride hydrolysis; this deficiency impairs triglyceride breakdown and disrupts phospholipid remodeling (84, 85). The downregulation of triiodothyronine also suppresses the expression of UCP1 in BAT, leading to a decrease in thermogenesis and energy expenditure. This facilitates the development of obesity (86). However, the effects of physiologically expressed and overexpressed NOD1 on thyroid hormone remain unknown. Further investigation is required to elucidate the mechanism by which NOD1 affects thyroid hormone homeostasis.

#### 5.2.5 Cell differentiation

The inflammation in the microenvironment inhibits the transcriptional activity of adipocyte differentiation regulators in adipose stem cells. Additionally, inflammation influences insulin signaling, impairing triglyceride synthesis and enhanced hydrolysis. Ultimately, the adipose differentiation in adipose stem cells is inhibited, while endothelial cell differentiation is promoted (87). It has been shown that activated NOD1 triggers activation of the NF-κB pathway in human adipose-derived stem cells and 3T3-L1 cells, leading to the inhibition of PPARγ and C/EBPα levels and attenuating adipocyte differentiation (88). The pro-cellular differentiation capacity of NOD1 has also been demonstrated in hematopoietic stem cells and mesenchymal stem cells (25, 89).

### 5.3 NOD1 and liver: inflammation, endoplasmic reticulum stress, insulin signaling

The liver is a vital organ responsible for metabolic and immune functions. Hepatic dysfunction contributes to dysregulation of glucolipid metabolism (90, 91).

Activated NOD1 increases the activation of NF-κB and MAPK pathways in hepatocytes, promotes the release of chemokines CCL5 and CXCL1 in an NF-κB-dependent manner, and together with cytokines such as IFN-γ mediates an increase in iNOS production and induces nitric oxide production (92). The pro-inflammatory response of NOD1 in the liver is affected by ER stress (55). ER stress facilitates the activation of NOD1 (20). Moreover, the concomitant occurrence of ER stress and NOD1 activation may synergistically exacerbate tissue inflammation.

Insulin inhibits gluconeogenesis and glycogenolysis while promoting glycogen synthesis and lipogenesis in hepatocytes (64). Similar to adipocytes, NOD1 interferes with insulin signaling in hepatocytes (42, 55, 66). NOD1 ligand reduces insulin-stimulated Akt phosphorylation in hepatocytes. Mice intervened with by NOD1 ligand have reduced insulin sensitivity and reduced phosphorylation of Forkhead-O1 in liver tissue (66). Forkhead-O1, a critical transcription factor for hepatic metabolism, promotes gluconeogenesis and affects intrahepatic lipid metabolism (93, 94). As a downstream target of the PI3K/Akt insulin signaling pathway, Forkhead-O1 phosphorylation is enhanced by Akt, leading to a reduction in its transcriptional activity, which in turn reduces the expression of genes associated with Forkhead-O1-induced gluconeogenesis (93, 94). It again demonstrates that NOD1 interferes with insulin signaling, reduces insulin sensitivity in peripheral tissues, and may decrease the inhibitory effect of insulin on gluconeogenesis.

In addition, NOD1-mediated inflammation and lipolysis products enhance hepatocyte lipid metabolism by enhancing cellular fatty acid uptake, mediating the expression of markers on the triglyceride synthesis pathway and output pathway, enhancing hepatocyte inflammation, and hindering insulin signaling (79). It suggests a remote regulatory role of NOD1 from adipocytes on hepatocytes.

### 5.4 NOD1 and cardiovascular system

The inflammatory mechanism of NOD1 hinders insulin signaling in the tissues, resulting in inefficient glucose utilization, being retained in the circulation and elevating blood glucose (67, 95). In response to abnormal blood glucose levels, the pancreas compensates by secreting more insulin, creating hyperinsulinemia (96). NOD1 promotes lipolysis, releasing inflammatory mediators and free fatty acids into circulation and enhances hepatic lipid metabolism (79). Finally, hyperglycemia (8) and high levels of insulin (97) are all involved in inducing activation of NOD1 in the cardiovascular system and triggering cardiovascular dysfunction (98).

#### 5.4.1 Inflammation

NOD1 is a key mediator of vascular inflammation at multiple sites throughout the body, with the cardiac macrovascular inflammatory response being the most intense (99). Activation of NOD1 induces multiple vascular inflammations throughout the body in mice, characterized by infiltration of neutrophils and macrophages mainly in the aortic root (99). This finding indicated the cardiovascular predisposition associated with NOD1-dependent vascular inflammation and the capacity of NOD1 to mobilize leukocyte subsets. The NOD1/RIP2/NF-κB signaling cascade in endothelial cells (ECs) upregulates VCAM-1 expression and promotes aggregation of monocytes and neutrophils in large vessels (100). Under HFD conditions, NOD1-deficient mice exhibit elevated serum levels of CCL2, CXCL1, and CXCL2, leading to the accumulation of immune cells in the circulation but reduced aggregation at the site of atherosclerotic lesions (56). These studies demonstrate that the recruitment of immune cells by NOD1 is closely related to its function in regulating chemokine expression. In addition, ECs (101–104), Vascular Smooth Muscle Cells (VSMCs) (97, 105), and cardiomyocytes (8) were all affected by NOD1-mediated inflammation. It can be reasonably assumed that persistent inflammation represents the primary mechanism through which NOD1 contributes to cardiovascular dysfunction.

#### 5.4.2 Cell transdifferentiation

Injury induces cellular reprogramming in tissues, causing cells with the ability to remodel to change their cellular identity known as cell trans-differentiation (106, 107). Endothelial-to-mesenchymal transition (EndMT) is a typical pattern of transdifferentiation characterized by decreased endothelial properties and enhanced mesenchymal properties (108). EndMT can be triggered by inflammation-related cytokines such as TGFβ, IL-33, IL-1β, TNF-α, etc., and mediated reprogramming by enforced transcription factors like Spi1 (109, 110). *In vitro* experiments have shown that NOD1 induces EndMT by activating the Akt/NF-κB pathway to create persistent inflammation in Human umbilical vein endothelial cells (111). This demonstrates that the inflammatory pathway downstream of NOD1 is involved in EndMT. Apart from inflammatory pathways, metabolic abnormalities such as hyperglycemia and oxidized OxLDL also activate EndMT (109). Interestingly, both activators of EndMT equally activate NOD1 (8, 100, 105), linking metabolic abnormalities, NOD1 activation, and cell trans-differentiation. Essentially, EndMT is a self-healing mechanism. However, in pathological states, it promotes endothelial dysfunction and contributes to pathological changes in cardiovascular diseases such as atherosclerosis, cardiac fibrosis, and pulmonary hypertension (108).

## 6 NOD1 and metabolic disease

### 6.1 Insulin resistance

Insulin resistance is a decrease in the sensitivity of insulin target tissues to insulin and the inability of normal levels of insulin to mediate appropriate levels of glucose-lowering action. Insulin resistance contributes to diseases such as obesity, type 2 diabetes, and metabolic syndrome (64, 68).

NOD1 inhibits the IRS and interferes with the PI3K/Akt pathway, which is the pathway by which insulin regulates metabolism (5, 6, 66, 67). This results in weakened insulin signaling in insulin-sensitive tissues. NOD1 mediates reduced glucose uptake and enhanced lipolysis in adipocytes, and adipose-secreting factors subjected to NOD1 enhance lipid deposition in hepatocytes (6, 79). NOD1 attenuates insulin-mediated phosphorylation of Forkhead-O1 in hepatocytes and may attenuate the inhibition of gluconeogenesis (66).

In addition, NOD1 expression in immune cells may contribute to insulin resistance. A clinical trial found that NOD1 and NOD2 mRNA expression in peripheral blood mononuclear cells from people with type 2 diabetes was positively correlated with insulin resistance and glycemic abnormalities (112). Moreover, NOD1 deficiency in immune cells protected mice from HFD-induced impairment of glycemic homeostasis. This may be attributed to reduced pro-inflammatory polarisation of macrophages in white adipose tissue and reduced recruitment of neutrophils (54).

### 6.2 Cardiometabolic disease

Metabolic abnormalities exacerbate cardiovascular damage and subsequently increase the risk of endpoint events in cardiovascular disease, a pathophysiological process in which metabolic risk factors and cardiovascular disease synergistically cause disease, also known as cardiometabolic syndrome or cardiometabolic disease (113, 114).

#### 6.2.1 Atherosclerotic lesions

Atherosclerotic lesions are characterized by inflammation and the deposition of lipid and fibrous substances in the intima of blood vessels (115, 116).

NOD1/RIP2/NF-κB pathway activation mediates endothelial inflammation and endothelial dysfunction, promoting the aggregation of leukocyte subsets into large blood vessels (56, 100). NOD1 activates EndMT (111). Fibroblast-like cells transdifferentiated from endothelial cells are involved in plaque formation. These fibroblast-like cells exhibit a phenotype characterized by reduced collagen expression and upregulation of destabilizing collagen-MMP expression. It suggests that EndMT enhances plaque instability (115). NOD1-deficient mice had reduced leukocyte subpopulations in the aortic root intima, decreased apoptosis, proliferation of VSMCs, and increased percentage of mature fiber type I collagen in the plaque, forming a solid plaque fibrous cap. It suggests that NOD1 activation promotes plaque generation and increases the risk of plaque rupture (105). Moreover, insulin resistance induces compensatory hyperinsulinemia (96). Activation of the NOD1 receptor in VSMCs by high concentrations of insulin increases the secretion of migration regulator IL-8 and inflammatory factor IL-1β in VSMCs (97). The migration of VSMCs to the intima enhances the accumulation of smooth muscle cells in atherosclerotic plaques (115).

The regulation of iron metabolism is likely one of the mechanisms through which NOD1 regulates atherosclerotic heart disease. Spleen, liver, and heart tissues of NOD1-deficient mice under HFD conditions exhibit significantly reduced iron content. In addition, genes related to iron metabolism show differential expression in the macrophages of these mice (117). In cases of iron deficiency, there is an increase in collagen fiber production (118), and increased macrophage glycolysis and lipid droplet. Iron overload favors M1-type differentiation (119). M1 macrophages amplify the inflammatory response at atherosclerotic sites and contribute to necrotic core formation and plaque instability (120).

#### 6.2.2 Diabetic cardiomyopathy

Diabetes mellitus mediates myocardial metabolic disorders, damages the cardiac microvascular circulation, causes cardiomyocyte dysfunction, promotes myocardial fibrosis, impairs systolic and diastolic function, and eventually leads to the development of congestive heart failure, a condition known as diabetic cardiomyopathy (121). NOD1 is capable of promoting the development of diabetic cardiomyopathy. The NOD1/NF-κB pathway and key proteins of apoptosis are highly expressed in heart tissues of type 2 diabetic mice (db/db mice), and the expression levels of key proteins in the NOD1/NF-κB pathway are further up-regulated after intervention with an activator for NOD1 (8). Meanwhile, activation of the NOD1/NF-κB pathway promotes activation of the TGF-β pathway in db/db mice cardiac fibroblasts (122). TGF-β is a cytokine involved in embryonic development and tissue repair. The activation and proliferation of fibroblasts are regulated by TGF-β, which promotes myocardial fibrosis (122). *In vitro* data also support that high glucose induces diabetic cardiomyopathy by stimulating the NOD1/NF-κB pathway, promoting cardiac cell apoptosis, and enhancing myocardial fibrosis (123).

#### 6.2.3 Blood pressure

Wistar rats with NOD1 agonist intervention showed increased expression of NOS2 and elevated nitric oxide production, resulting in lower blood pressure by vasodilation. The rats also exhibited symptoms of tachycardia, impaired renal function, and stimulated coagulation. Additionally, the aorta incubated with NOD1 agonist demonstrated reduced response to vasopressin (124). *In vitro* experiments with NOD1 similarly revealed enhanced nitric oxide release from VSMCs (125).

Different from Wistar rats (124), spontaneously hypertensive rats (SHRs) exhibit endothelial dysfunction (126). As SHRs age, there is an increase in blood pressure levels, myocardial NOD1/RIP2 expression levels, and myocardial remodeling levels (127). NOD1 inhibitor delayed vascular remodeling in SHRs (127, 128). The activation of NOD1 in the cardiovascular system promotes inflammation, induces EndMT (111), and increases angiogenesis in ECs (129). Multiple pathophysiological mechanisms jointly mediate myocardial and vascular remodeling and affect blood pressure levels (130).

## 7 Inhibition of NOD1-mediated metabolic damage

### 7.1 Interfering with NOD1 signaling

Previous studies have found that HFD and saturated fatty acids contribute to NOD1 activation (41, 62). Docosahexaenoic acid is an n-3 polyunsaturated fatty acid that can be obtained from marine fish and fish oil (131). It inhibits cellular inflammation mediated by lauric acid and iE-DAP (62). Need to verify if it has the same NOD1 inhibitory function *in vivo*.

Troxerutin, commonly found in the daily diet, is a hydroxyethylated compound of rutin, a natural flavonoid glycoside. It has been proven to have therapeutic effects on metabolic syndromes such as diabetes and cardiovascular disease (132). It has the efficacy of inhibiting ER stress, regulating the NOD1 pathway, and inhibiting HFD-enhanced hepatic gluconeogenesis (55).

Several natural monomers/extracts have been reported to ameliorate NOD1-mediated metabolic damage. Ginsenoside Rg3 (Rg3), an active ingredient in red ginseng, has been shown to inhibit intimal hyperplasia caused by inflammation. Rg3 inhibits NF-κB nuclear translocation and suppresses Akt/NF-κB signaling by up-regulating miR-139-5p expression, contributing to the amelioration of EndMT in human umbilical vein endothelial cells induced by NOD1 (111). Moreover, purple sweet potato pigments ameliorate hepatic inflammation induced by an HFD (133), and Osthole exhibits cardioprotective effects (134). These effects are probably related to the inhibition of NOD1 and downstream pathway activation. However, further research is required to confirm the mechanism of action.

Tauroursodeoxycholic acid (TUDCA), a hydrophilic bile acid with a cytoprotective effect, has been approved for clinical use in the treatment of primary biliary cholangitis (135). It inhibits ER stress and the NOD1 pathway, improving glucose metabolism in HFD-fed mice (55).

PPARγ is a ligand-activated transcription factor that is highly expressed in adipocytes (136). PPARγ reduces the inhibitory effect of NOD1 on adipose tissue browning during adipocyte differentiation (82). It also regulates the expression of miR-125a, which inhibits the expression level of NOD1 and its pro-inflammatory and pro-angiogenic function in ECs (129). Thiazolidinediones are PPARγ receptor agonists that increase insulin sensitivity (136). However, thiazolidinediones have not been studied to see how this affects the NOD1.

The Nuclear factor of activated T cells (NFAT) is highly expressed in monocytes of individuals diagnosed with type 2 diabetes and is positively correlated with NOD1, insulin resistance, and blood glucose levels (97). NFAT regulates NOD1 transcription in VSMCs, promotes cell proliferation, and induces phenotypic transformation of VSMCs under hyperinsulinemic conditions (97). Targeted inhibition of NFAT can regulate the NOD1 pathway and alleviate the proliferation and differentiation of VSMCs. However, NFAT consists of five subtypes, each with different structures and functions (137). It is necessary to explore the subtypes of NFAT associated with NOD1 and verify the efficacy and safety of selective inhibition.

### 7.2 Targeting RIP2

Another strategy is to target RIP2 and interfere with NOD/RIP2 signaling. Tyrosine kinase inhibitors (TKIs) are currently in clinical use for cancer treatment. Some TKIs can control blood glucose but the mechanism is unclear (138). TKIs targeting RIP2 may ameliorate NOD1-mediated metabolic abnormalities (138). For example, gefitinib, which has been used in the clinic, reduced NOD1 ligand-induced metabolic damage in both cellular and animal experiments (138). However, because of the inhibitory properties of TKIs on a variety of kinases, multiple potential mechanisms exist to ameliorate metabolic abnormalities (139). NOD1/RIP2 is not a critical metabolic regulatory pathway for gefitinib (139). The effect and mechanism of the inhibitory effect of gefitinib on the NOD1/RIP2 pathway may require further evaluation.

RIP2 is a downstream signal molecule common to NOD1 and NOD2 (140). Considering that the two have opposite functions on insulin sensitivity (140), direct inhibition of RIP2 may reduce the glucose-lowering effect of NOD2. Furthermore, it is not known whether direct inhibition of NOD1 has an effect on the body's immune function. Perhaps being able to target NOD1 expression in target tissues is a better strategy.

## 8 Risk assessment indicators for cardiometabolic disease

In a study, NOD1 has been used as one of the risk prediction classifiers for acute myocardial infarction associated with cellular pyroptosis. Due to the small sample size of this study, future validation through *in vivo*, *in vitro*, and clinical trials is needed (141).

Mechanistically, NOD1 disrupts insulin signaling and mediates insulin resistance (5, 6, 66, 67). In addition, NOD1-induced inflammation mediates EC, VSMC, and cardiomyocyte dysfunction. Insulin resistance increases the risk of cardiovascular disease (142). It has been found that people with the NOD1 (Glu266Lys) Lys/Lys genotype are affected by saturated fatty acids, increasing the risk of insulin resistance (60). We envisage that NOD1 has the potential to contribute to the assessment of the risk of cardiometabolic diseases associated with endothelial damage such as atherosclerosis. This is particularly applicable to insulin-resistant populations or those with a Lys/Lys genotype with NOD1 (Glu266Lys). Considering that the mechanism by which NOD1 mediates atherosclerosis is related to its function in promoting the aggregation of leukocyte subpopulations (100), assessing NOD1 expression on leukocyte subpopulations in the peripheral circulation of subjects may be a feasible approach.

## 9 Summary

NOD1 is involved in endocrine and metabolic processes in several tissues throughout the body. As a sensitive intracellular sensor, NOD1 can rapidly recognize host microbial signals, sense disturbances in the intracellular environment, and transmit downstream signals. NOD1 induces oxidative stress, ER stress, EndMT, and apoptosis to respond to the challenges. Inflammatory response is the most central mechanism of NOD1. Under physiological conditions, NOD1 functions in immune defense, determines cell differentiation direction, promotes hormone secretion, and maintains internal environment stability. Under pathological conditions, NOD1 is abnormally expressed, leading to metabolic dysfunction in tissues and organs. NOD1 triggers abnormal cellular metabolism, including glycolipid metabolism, and is involved in the development and progression of insulin resistance and cardiometabolic disease. NOD1 not only increases cardiovascular disease risk factors, but also mediates damage to cardiovascular endothelium, vascular smooth muscle, and myocardium. Therefore, NOD1 has the potential to be an indicator for assessing cardiometabolic diseases. There are several issues for further study (1) The effect of NOD1 on pancreatic islet function under conditions where abnormalities of glucolipid metabolism have already occurred. (2) Whether NOD1 interferes with other insulin receptor substrates. (3) Signal transduction between NOD1 signaling downstream and insulin signaling. (4) Further studies are needed to investigate the metabolic role of NOD1 in liver and skeletal muscle in physiopathological states.
